# Esthetic Solutions With Layered Zirconia Prostheses: A Case Report

**DOI:** 10.7759/cureus.49822

**Published:** 2023-12-02

**Authors:** Mithilesh M Dhamande, Arushi Beri, Anjali Bhoyar, Surekha A Dubey, Seema Sathe

**Affiliations:** 1 Prosthodontics, Sharad Pawar Dental College and Hospital, Datta Meghe Institute of Higher Education and Research, Wardha, IND

**Keywords:** emax, prosthodontics, layered dental zirconia, fixed dental prosthesis, anterior aesthetics

## Abstract

To meet the challenges of rehabilitating anterior teeth and achieving natural-looking restorations, it is essential to address issues such as improper shape, size, irregular gingival contour, and unaesthetic shades. The increasing demand for aesthetically pleasing and metal-free solutions has popularized materials like dental zirconia, offering optimal aesthetics and desirable mechanical properties. Within this context, a case report highlights clinical experiences with layered zirconia fixed dental prostheses designed specifically for anterior teeth. The report focuses on the prosthetic rehabilitation of both endodontically treated and vital abutments, exploring the influence of zirconia composition, design, layering technique, shade selection, occlusion, and the unique clinical challenges associated with each condition. The selection of zirconia composition, framework design, and shade in layered zirconia prostheses is intricately tied to the characteristics of the abutments. This interconnectedness underscores the importance of a thoughtful and customized approach to address the specific requirements of each clinical scenario.

## Introduction

The pursuit of esthetic excellence is a core objective in restorative dentistry, with dental ceramics being a longstanding choice for their superior properties and natural tooth-like appearance. Zirconia, in particular, has gained widespread acceptance as an aesthetically pleasing material in dentistry [1]. Traditional fixed dental prostheses often utilize a zirconia substructure with veneering porcelain; however, issues such as breakage, fracture, delamination, and chipping have led to a notable shift toward monolithic zirconia crowns. Monolithic zirconia crowns, renowned for their full contour design, have seen increasing popularity owing to their numerous advantages. This preference for monolithic zirconia crowns is particularly significant in the case of maxillary anterior teeth, where achieving optimal aesthetics and functionality is paramount. A detailed case study is presented here, showcasing the successful treatment of a teacher using zirconia all-ceramic crowns [2-5]. The achieved outcome not only meets rigorous aesthetic standards but also ensures full functional competence, resulting in a positive psychological and mental impact on the patient. It is crucial for the dentist involved in crafting ceramic crowns to be sensitive to the patient's esthetic preferences, as technical accuracy alone may not suffice to fulfill individual symmetry and aesthetic desires. Achieving successful results requires a thorough understanding of the patient's expectations as well as careful consideration of anatomical considerations. The use of CAD/CAM (computer-aided design CAD and computer-aided manufacturing CAM) zirconia material in treatment is also covered in the case study, with special attention to a patient who had several discolored fillings in their maxillary anterior teeth. The paper emphasizes how zirconia is becoming more and more important in modern dentistry procedures [6-8].

## Case presentation

**Figure 1:**
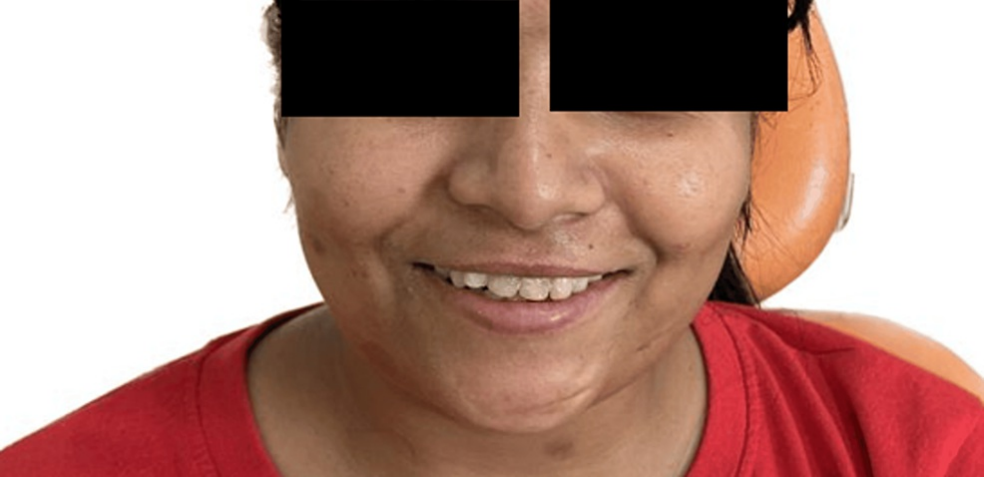
Pretreatment photographs

The case presented involves a 32-year-old female teacher seeking a durable esthetic solution for discoloration and multiple fillings in her maxillary anterior teeth, as depicted in Figure 1.

**Figure 2:**
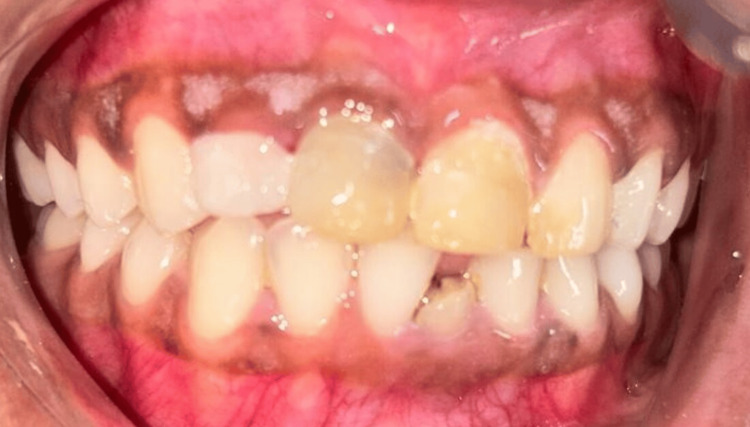
Pretreatment intra-oral photograph

**Figure 3:**
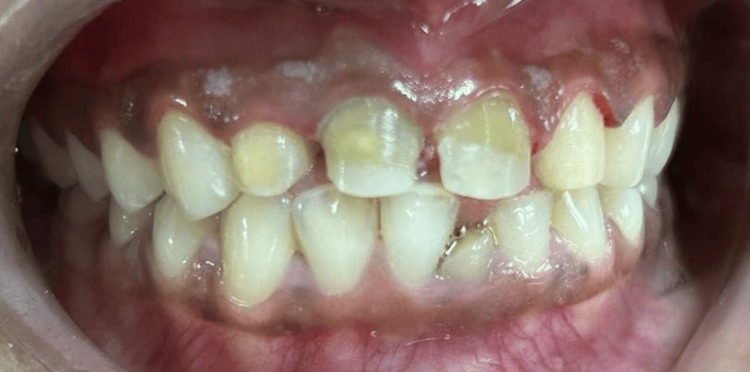
Tooth preparations with 12, 11, 21, 22

A thorough assessment, including detailed case history and extra-oral, intraoral, clinical, and radiographic examinations, revealed generalized discoloration and previous composite restorations. The vitality pulp test confirmed the necessity for elective root canal treatments, and various treatment modalities were discussed with the patient. After root canal therapy, a thorough treatment plan was started with the patient's consent, which included zirconia crowns for the anterior maxillary teeth. Multiunit composite restorations were discovered during intraoral exams. A preapical radiography analysis revealed lamina dura loss and periodontal ligament enlargement. Maxillary and mandibular imprints were taken at the beginning of the procedure, and any existing restorations were then scaled, polished, and enhanced. A diagnostic wax-up was collaboratively prepared with the dental technician. Composite resin was used to shape the maxillary incisors; shade C1 was used. In addition to elective root canal therapy, tooth preparations were carried out (Figure 3), and FRC posts were placed into the roots.

**Figure 4:**
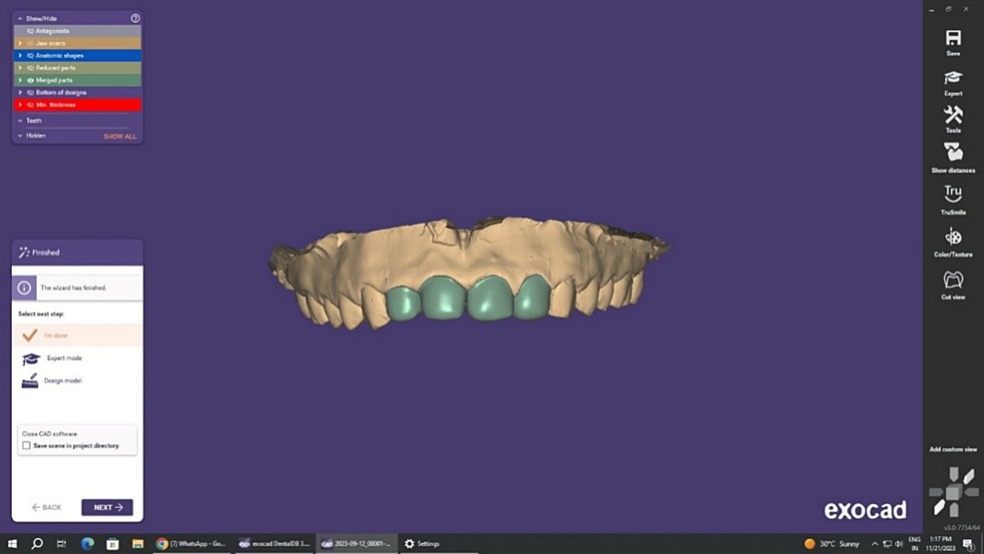
Designing of zirconia coping with exocad software

Cores were built using composite resin, with the finish line placed slightly below the gingiva. During impression recording, a retraction cord was positioned in the buccal gingival sulcus, and full-arch impressions were crafted using polyvinyl siloxane and the putty reline technique. The impressions were then poured into the dental laboratory, where CAD-CAM FSZ Zirconia coping was performed (Figure 4).

**Figure 5:**
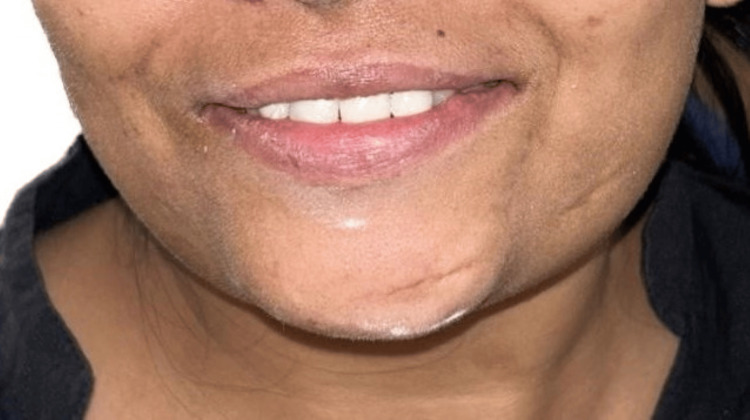
Post-op picture

Dentsply Sirona was meticulously fabricated, and layering of zirconia copings was done with emax (Ivoclar Vivadent). The final visit involved a porcelain try-in, interocclusal adjustment, and confirmation of canine guidance and movements before glazing. Glazed zirconia crowns were subsequently cemented. The entire process, from construction and production to cementation, strictly adhered to the manufacturer's instructions (Figure 5 shows the post-op picture).

A follow-up program was established to assess the longevity and success of the all-ceramic zirconia crowns. This case underscores the importance of meticulous planning and execution in achieving both aesthetic and functional goals in dental rehabilitation.

## Discussion

A tooth-like translucent look, high biological compatibility in direct contact with oral tissues and the periodontium, and a wear pattern that closely resembles real tooth enamel are just a few of the exceptional features of CAD/CAM zirconia ceramic prostheses [9]. This positions them as a preferable choice, especially when compared to lithium silicate ceramics, which, while versatile, may not offer the same range of indications, particularly for larger restorations in the posterior region. Notably, the most prevalent Vertucci classification type in maxillary central and lateral teeth was found to be type I, which denotes a single canal with a single root and canal structure. Variances beyond type I were also noted, though, and these variances complicate the elective root canal procedures for these teeth [10-13]. The evolution of zirconia ceramics, particularly with the introduction of fifth-generation mixed zirconias, has broadened their indication range and led to the availability of numerous material variants tailored for individualized applications. The fifth-generation mixed zirconias offer a diverse range of indications, extending from small three-span bridges in the anterior region to the approval of larger 14-unit bridges, allowing for versatile use in dental restorations. The behavior of front maxillary teeth repaired with CAD/CAM zirconia crowns following root canal therapy showed an equal or greater survival rate compared to alternative all-ceramic materials, such as porcelain fused to metal and e.max ceramic, in a clinical and radiographic investigation [14]. The clinical outcomes for the patient in this case encompassed the effective masking of discoloration with durable and biocompatible crowns. The color matching not only addressed aesthetic concerns but also contributed to boosting self-esteem and enhancing social communication, particularly in the patient's role as a schoolteacher. The harmony in the smile line and arrangements during smiling and speaking further underscored the success of the treatment in meeting both functional and aesthetic objectives [15-20].

## Conclusions

This case report offers comprehensive insights into the restoration of anterior fractured and discolored teeth. Emphasizing the crucial principle of conserving the remaining tooth structure in dentistry, the fractured tooth underwent root canal treatment and composite buildup, achieving successful restoration. Considering the patient's priority for aesthetics, the treatment of choice involved an all-ceramic restoration, specifically a layered zirconia restoration. Zirconia restorative prostheses made using CAD/CAM technology exhibit superior biocompatibility, which leads to reduced wear on neighboring teeth and long-lasting aesthetics with reliable color stability. These characteristics help patients regain a full social life and raise their confidence and self-worth. In prosthodontics, zirconia-based restorations show great promise because of their exceptional mechanical, chemical, and clinical performance.
